# Self-limiting population genetic control with sex-linked genome editors

**DOI:** 10.1098/rspb.2018.0776

**Published:** 2018-07-25

**Authors:** Austin Burt, Anne Deredec

**Affiliations:** 1 Life Sciences, Imperial College, Silwood Park, Ascot SL5 7PY, UK; 2 UMR BIOGER, INRA AgroParisTech, Université Paris-Saclay, Avenue Lucien Bretignières, 78850 Thiverval-Grignon, France

**Keywords:** pest control, sterile insect technique, gene drive, gene editing, CRISPR, Y-linked editors

## Abstract

In male heterogametic species the Y chromosome is transmitted solely from fathers to sons, and is selected for based only on its impacts on male fitness. This fact can be exploited to develop efficient pest control strategies that use Y-linked editors to disrupt the fitness of female descendants. With simple population genetic and dynamic models we show that Y-linked editors can be substantially more efficient than other self-limiting strategies and, while not as efficient as gene drive approaches, are expected to have less impact on non-target populations with which there is some gene flow. Efficiency can be further augmented by simultaneously releasing an autosomal X-shredder construct, in either the same or different males. Y-linked editors may be an attractive option to consider when efficient control of a species is desired in some locales but not others.

## 1. Introduction

The most widely used genetic approach to pest control thus far has been the mass release of sterile males [1]. This approach requires inundating the target population with males that have been sterilized by radiation, infected with *Wolbachia* or modified by transgenesis [2], and has been used successfully against some agricultural pests and disease vectors. However, the numbers released typically have to be at least 10-fold larger than the target population, and sustained for multiple generations, and the associated costs of mass production limit the range of species for which this approach is suitable.

In principle, the introduction into populations of genetic constructs showing preferential inheritance (gene drive) may be substantially more efficient than inundative approaches like the sterile insect technique, requiring only small inoculative releases to suppress a population (e.g. less than 1% of the target population) [3,4]. Promising proof-of-principle demonstrations of such constructs have been reported for malaria-transmitting mosquitoes [5–7]. One reason for the predicted efficiency is that natural processes of dispersal and migration can be exploited to introduce the construct (and its suppressive effect) from the population into which it was released into other populations that may be more difficult to access [8,9].

In some cases it may be desirable to control a pest species in one location but not in another—for example, to control an agricultural pest in a farmer's field but not in a nature reserve, or to control an invasive species where it is invasive but not in its native range. In these cases, depending on the biology of the species involved, one may want to consider interventions that are more efficient than mass release of sterile males, but will not have an appreciable impact on non-target populations, even if there is some gene flow. Possibilities include releasing males that carry constructs that kill female descendants [10] or cause them to have predominantly male offspring [5], both of which can, in some circumstances, be more efficient than release of sterile males, and are still expected to have geographically restricted impacts. If the goal is to modify the target population rather than suppress it, then there is a range of options, some of which should allow geographical targeting [11,12].

In this paper, we explore the possibility of using a construct inserted on the Y chromosome to make edits in autosomal or X-linked genes needed for female survival or reproduction. The key idea is that the construct acts in males to reduce the fitness of female descendants, but because the Y is not found in those descendants, the construct will not be selected against by the harm it causes. This idea was previously suggested by Deredec *et al*. [13] as a way to increase the efficacy of a driving Y chromosome, but clearly it can also be used in a non-driving context. We use relatively simple ‘strategic' models to explore some of the features of this approach, starting with an idealized case and comparing its efficiency with that of other self-limiting constructs. We then examine the impacts of various deviations from the ideal, to assess the robustness of the approach, and the impacts of gene flow both into and out of the target population. Finally, we explore how efficiencies can be further increased by including another transgene in the releases to boost the frequency of the modified Y, and discuss some of the molecular options for building these constructs. For ease of exposition, we emphasize results from a set of exemplar scenarios that we consider most illuminating, rather than an exhaustive analysis of a simple model that would need to be made more realistic for any particular use case. Our modelling demonstrates that Y-linked genome editors (YLEs) have a unique combination of features and should be a useful addition to the menu of options to be considered in designing and developing pest control programmes.

## 2. Results

### (a) Ideal case and comparison with alternatives

We first consider the release of males carrying an idealized construct in which the only effect of the YLE is to induce 100% knock-out of a target gene, which may be either autosomal or X-linked, and the only effect of the knock-out is complete dominant female lethality. For comparison, we also model idealized versions of several other strategies, including release of males homozygous for:

(1) A dominant autosomal lethal gene; this is the bisexual RIDL (bi-RIDL) approach and, at least in our model, is equivalent to the release of radiation-sterilized males or of males carrying a *Wolbachia* strain that renders them incompatible with the target population [14–16].
(2) A dominant autosomal female-specific lethal gene (fs-RIDL); males inheriting the gene have normal fitness and transmit it to the Mendelian 50% of progeny [17,18]. In some species it might be possible to use repressible autosomal copies of a male-determining gene for this strategy [19,20].
(3) A dominant autosomal female-specific lethal gene showing drive in males (fs-RIDL-drive); males inheriting the gene have normal fitness and transmit it to 100% of progeny [7,18]. Possible implementations include using a constitutively active homing construct to target a gene essential for female development [21,22] or linking a male-determining gene to a male-limited gene drive system [23,24].
(4) A dominant autosomal gene causing all sperm to carry the Y chromosome, and therefore all progeny to be male (X-shredder) [5,6,25].

The efficiency of these alternative control strategies was compared using a simple deterministic model of a population with discrete non-overlapping generations, random mating, male heterogamety, separate juvenile and adult stages, and density-dependent mortality occurring in the juvenile stage only. Males are assumed not to be limiting in the production of fertilized eggs (see electronic supplementary material for further details on the model). For the YLEs and alternatives (1)–(3), we considered variants where the lethal gene acts before density-dependent mortality (e.g. at the embryonic stage) or after (e.g. at the juvenile-adult transition), and for all of these except (1) we also considered the case where the gene causes female sterility rather than death, as such differences have previously been shown to impact the efficiency of control [13,26–28]. In all cases, we assumed that only adult males are released, that they have survival and mating success equal to the wild-type males, and that a constant number is released each generation (rather than, for example, a constant proportion of a declining target population).

**Figure 1.**
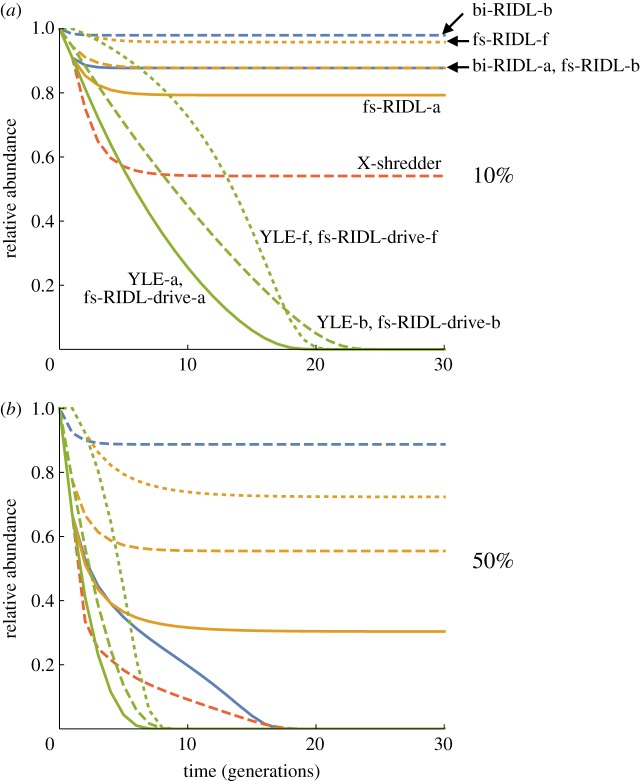
The time course of population control with different self-limiting constructs. The number of females in the population, relative to the pre-release number, is plotted against the number of generations of releases. In each generation an equal number of males is released, either (*a*) 10% or (*b*) 50% of the initial male population. Alternative male genotypes include bi-RIDL (blue), fs-RIDL (orange), X-shredder (red), and fs-RIDL-drive and YLE (indistinguishable, green), and alternative modes of action include death after density-dependent mortality (suffix -a, solid lines), death before density-dependence (-b, dashed) or female sterility (-f, dotted). *R_m_* = 6.

Figure 1 shows the population size (number of females) over time with recurrent releases of the different alternative constructs into a target population with an intrinsic rate of increase of *R_m_* = 6 and release rates of 10% or 50% of the initial male population size. In both cases the different types of YLE eliminated the population, with no difference whether the target gene was autosomal or X-linked, and the most rapid decline occurring if the edit caused death after density-dependence (hereafter denoted a YLE-a construct). Constructs that combined dominant female lethality and male drive were equally good as the YLEs (indeed, indistinguishable), and all other approaches were less effective.

**Figure 2.**
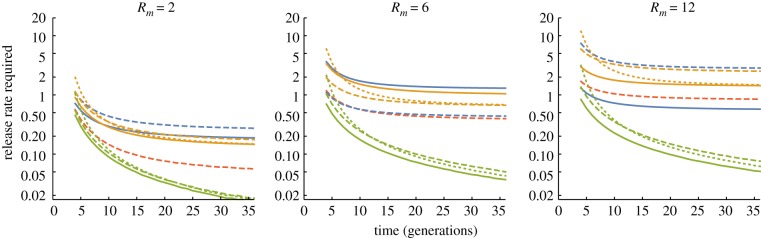
Release rates required to suppress the number of females in a target population to 5% of its initial value as a function of the numbers of generations of releases. Each line represents an alternative strategy; colour and style of lines as in figure 1. Release rates calculated as a proportion of the initial number of males; note log scale.

One measure of the efficiency of a construct is the release rate required to achieve a specified level of control in a specified time frame. Figure 2 shows the release rates required to suppress the number of females in a population by 95% as a function of the duration of the intervention programme for the different alternative constructs and for target populations with different intrinsic rates of increase (*R_m_*, defined as the expected number of daughters produced by a female in the absence of density-dependent mortality). As expected, the required release rates decline as the allowed number of generations increases, and larger releases are needed when *R_m_* is higher. Again, YLEs and female lethal/male drive constructs were indistinguishable, and consistently more efficient than the alternatives, with the differences increasing with both the duration of control and *R_m_*. For a YLE-a construct, the required release rates for different levels of suppression and different values of *R_m_* are shown in table 1, assuming 36 generations of releases. In the easiest scenario considered (67% suppression with *R_m_* = 2), release rates of only 1% of the initial population are needed, whereas for the most difficult scenario (99% suppression with *R_m_* = 12), release rates of 5.8% are needed. Sterile male releases would need to be one or two orders of magnitude larger (table 1).

**Table 1.** Release rates required to control a population with a Y-linked editor as a function of the desired level of suppression and the intrinsic rate of increase of the population (*R_m_*). Release rates are for the idealized case with the target gene causing female-specific lethality after density-dependence, and assuming releases occur for 36 generations. Numbers in parentheses indicate how many times larger the release rate would have to be to achieve the same level of control with sterile males (bi-RIDL-b).

level of suppression (%)	*R_m_*		
	2	6	12
67	0.010 (27)	0.021 (58)	0.026 (108)
95	0.015 (18)	0.036 (35)	0.050 (56)
99	0.016 (16)	0.041 (32)	0.058 (49)

### (b) Sensitivity to deviations from ideal case

**Figure 3.**
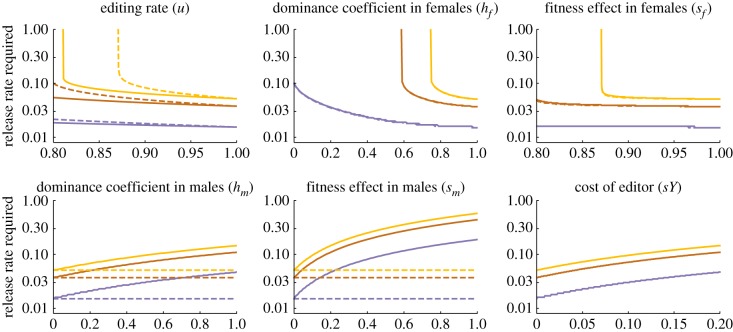
Release rates required to suppress the number of females in a target population to 5% of its initial value in 36 generations with a YLE-a construct as a function of various genetic and fitness parameters, for *R_m_* = 2, 6, and 12 (blue, brown and yellow, respectively). Solid lines are for an autosomal target locus, and dashed lines for X-linked (when not visible, they are indistinguishable from the solid lines). When varying *h_m_*, *s_m_* was set to 0.2, and when varying *s_m_*, *h_m_* was set to 1; otherwise, all parameters set to baseline ‘idealized' values. Release rates higher than those shown always give greater suppression after 36 generations, except when the target is an autosomal locus and the editing rate (*u*) less than 1—in this case higher release rates can give an initially faster decline, but a higher equilibrium number of females.

The idealized parameter values we have considered thus far may be difficult to achieve in practice, and therefore it is important to consider how efficiency will be affected by less than perfect performance. As an example, figure 3 shows how the release rates required for 95% suppression in 36 generations are affected by changes in six molecular and fitness parameters, leaving the others at their baseline values. Release rates are not much affected by decreasing the knock-out rate, as long as it remains above 0.9, and not much affected by decreasing the dominance coefficient of female lethality, or the homozygous effect on female survival, as long as they remain above 0.8. If the parameters fall much below these thresholds, then population control may fail, particularly for higher *R_m_*. It makes little difference in these cases whether the target gene is X-linked or autosomal. By contrast, if the knock-out also affects male fitness, then the required release rates increase if the target gene is autosomal but not if it is X-linked. Males with the YLE transmit X-linked targets only to their daughters, and if those die, the edit does not appear in male descendants, so male fitness effects do not matter. Finally, if the YLE itself reduces male fitness, then release rates will need to increase.

### (c) Effect of halting releases

**Figure 4.**
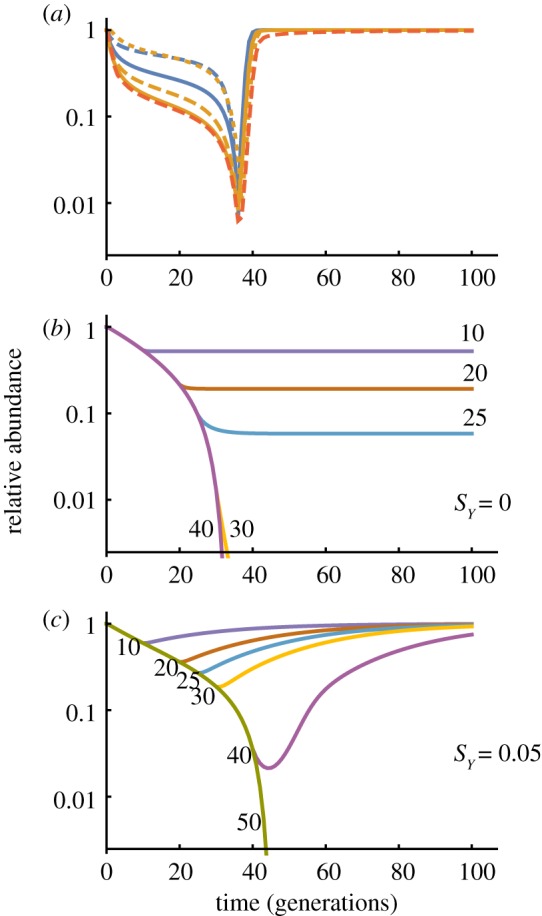
The effect of halting releases. (*a*) Relative abundance of females after 36 generations of releases of bi-RIDL, fs-RIDL or X-shredder constructs. Colour scheme as in figure 1. For each construct the release rates were chosen to give approximately 99% maximum suppression. In all cases populations recover rapidly after releases stop. (*b*,*c*) Relative abundance of females after releases of YLE-a constructs with releases stopping after the indicated number of generations with (*b*) *sY* = 0 or (*c*) *sY* = 0.05. Release rates are 0.05 with an autosomal target gene.

Because selection against YLEs is absent or weak, the consequences of stopping releases before population elimination are markedly different than with the other self-limiting strategies, where the population rapidly recovers (figure 4*a*). In the idealized case of a YLE with no effect on male fitness, halting releases stops the population decline, but it does not recover, and the population remains suppressed indefinitely (figure 4*b*). Alternatively, if the YLE imposes a small fitness cost on males (*s_Y_* > 0), then it is slowly lost after releases are stopped, and the population slowly recovers (figure 4*c*).

### (d) Effect on non-target populations

We now consider two populations, one targeted for control and the other not, with some migration between them. Obviously, immigration of wild-type individuals into the target population will increase the release rates required to achieve control, particularly if females mate before immigrating [29] (electronic supplementary material, figure SI-1). To analyse the impact of gene flow from the target to non-target populations, consider first the number of transgenics in the target population. For example, a release rate of 3.7% of an ideal YLE-a for 36 generations will suppress a population by 95% (table 1). The maximum number of YLE males in any generation is about 0.5, and the total number of YLE males over the 36 generations is about 13 (i.e. 13 times the original number of males; electronic supplementary material, figure SI-2). If, for example, the probability that any one of these males emigrates to and reproduces in the non-target population is 10^−3^, then the total number of male emigrants over the 36 generations is 0.013 (i.e. 1.3% of the original population), with a maximum value of 0.0005 in any one generation. The expected proportion of YLE males in the non-target population will then depend on its size relative to the target population. Assuming equal population sizes, the YLE is expected to be in 1.4% of males after 50 generations, and the number of females to be 98.3% of the number pre-release. If there is a small cost to the YLE (e.g. *s_Y_* = 0.05), and release rates increased to 5.3% to still achieve 95% suppression of the target population in 36 generations, then impacts on the non-target population are even lower, with a frequency in males after 50 generations of 0.4% and a female abundance of 99.5% (electronic supplementary material, figure SI-2). Tolerance thresholds for spread to neighbouring populations will be determined on a case-by-case basis.

### (e) Augmenting transmission of the Y-linked genome editor

**Figure 5.**
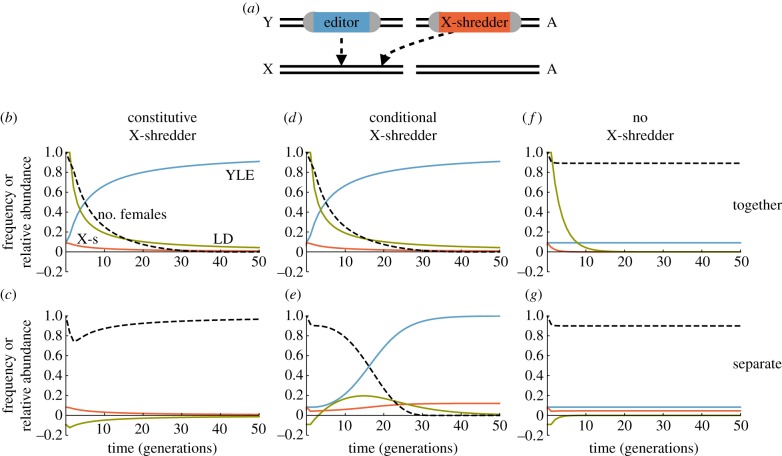
(*a*) Modelled strategy in which a YLE targets an X-linked female fertility gene and an autosomal (A) X-shredder cleaves the X chromosome during gametogenesis, causing preferential transmission of the Y chromosome to the next generation. (*b*–*g*) Impact of including an autosomal X-shredder in the releases. Scenarios considered include the X-shredder being present in the same released males as the YLE (*b*,*d*,*f*), or in additional males that carry a wild-type Y (*c*,*e*,*g*), and an X-shredder that causes 100% transmission of the Y regardless of the genotype of the Y (*b*,*c*), or only of those carrying the YLE (*d*,*e*). (*f*,*g*) shows dynamics for a non-functional X-shredder (no impact on Y transmission). In all cases there is a single 10% release in generation 0 of males carrying the YLE; in the ‘separate' scenarios there is an additional 10% release of males carrying the X-shredder. Black dashed lines shows abundance of females over time; blue line the frequency of the YLE in males; red line the frequency of the X-shredder in males; and green line the correlation coefficient among males between the presence of the YLE and of the X-shredder (heterozygotes and homozygotes combined). All parameters at idealized values.

All else being equal, the higher the frequency of the YLE in a population, the greater the load it imposes upon it. Is there any way to increase this frequency without releasing more males? In principle a YLE could be put on a driving Y chromosome, as modelled by Deredec *et al*. [13], but then it may spread to and substantially suppress geographically distinct populations if there is even infrequent gene flow. Here we consider the possibility of releasing a YLE along with an autosomal X-shredder or other gene that leads to disproportionate transmission of the Y chromosome (figure 5*a*). Four possible implementations are considered, including an X-shredder that is either constitutive or conditional (i.e. causes biased transmission of all Ys or just of YLE-bearing Ys), and that is released either in the same males as the YLE or in different males released at the same time. In each case the YLE targets an X-linked locus, and we initially model the impact of a single one-time release of 10% of the pre-release population.

Exemplar results for the four idealized scenarios are shown in figure 5, along with no X-shredder release for comparison. With a constitutive X-shredder, if it is released in the same males as the YLE, it boosts the transmission of the YLE from one generation to the next, which therefore continues to increase in frequency even after releases have stopped (figure 5*b*, blue line), and as a result the population size continues to fall (black dashed line). The boost occurs because of the positive association, or linkage disequilibrium, between the X-shredder and the YLE (green line), which starts complete and then gradually decays over time, so the rate of increase of the YLE also slows. By contrast, if there are separate releases of YLE males and X-shredder males, then there is initially a negative correlation between the two loci, the X-shredder boosts the transmission of wild-type Ys, the frequency of the YLE declines over time, and the final level of control is worse than if no X-shredders were released (figure 5*c* versus figure 5*f,g*).

If instead the X-shredder only acts in the presence of the YLE, then it can boost the frequency of the YLE and the impact of a release whether it is initially in the same or different males, though the dynamics are different in detail (figure 5*d*,*e*). If they are released in the same males, then with the idealized parameter values considered here there is no difference in the dynamics whether the X-shredder is constitutive or conditional, because a 100% effective X-shredder is always transmitted to males, and remains associated with the Y chromosome with which it was released. Differences between constitutive and conditional X-shredders would appear with less than perfect performance. By comparison, if the YLE and X-shredder are released in separate males, then, at least in this example, the level of suppression is initially lower, but later it is higher (figure 5*d*,*e*). Importantly, in all these scenarios, although the YLE can substantially increase in frequency after release, the X-shredder does not, indicating that it may be unlikely to spread much into other geographically distinct populations, though a detailed analysis is beyond the scope of this study.

As the inclusion of an X-shredder causes the frequency of the YLE and the extent of suppression to continue to increase after a release, it may be more efficient to release once in a population rather than repeatedly. If we now ask how large a single release must be to achieve 95% suppression in 36 generations, it turns out to be 6.0% if the YLE-a and X-shredder are released in the same males, and 5.4% if the X-shredder is conditional and is released separately (i.e. 5.4% of YLE-a males and 5.4% of X-shredder males, assuming equal releases of the two types). In either case the total number of males that need to be released is more than an order of magnitude lower than without an X-shredder (3.6% releases for 36 generations; table 1).

## 3. Discussion

Our modelling has shown that YLEs can give substantially more efficient control of populations than the sterile insect technique or many other proposed variants, while still allowing the impact to be focused on a particular geographical region. This efficiency derives from the fact that YLEs are not selected against as a consequence of the harm they cause—they are evolutionarily insulated from their impacts [13]. YLEs share this feature with the ‘Trojan female' proposal of releasing into populations mitochondria that specifically impair male fertility [30]. Along similar lines, some naturally occurring maternally inherited endosymbionts in insects have evolved to kill male embryos, to free up resources for their sisters [31], which highlights an important assumption of our models: that the YLE does not confer a fitness advantage to the male. Such an advantage could occur if brothers and sisters compete more intensely than random and the YLE caused early (e.g. embryonic) death of females. In such a situation the YLE could show a form of drive [32] and spread in a self-sustaining way, including to neighbouring populations.

Other types of sex-linked genome editors are also possible and may be useful. We have considered the most obvious classes of target gene (lethals and steriles), but in some species there may be other possibilities, such as edits that convert females into males. In species where males play a more important role in provisioning for the next generation, or otherwise are more harmful, then it may be worth considering an X-linked editor that targets a Y-linked gene, including a male-determining gene. And in female heterogametic species, where females are WZ and males ZZ, if it is still desirable to release only males, then a Z-linked editor that targets a W-linked locus (including a female-determining gene) could be considered. In species that do not have differentiated sex chromosomes but have a male-determining locus (e.g. some culicine mosquitoes), the editor could be inserted near the latter.

Our primary metric for the efficiency of an intervention strategy has been the release rates required to achieve a particular level of control. We have assumed the released males are equally fit as the wild males, though differences could arise because the released males are of lower vigour (e.g. due to poorer rearing environment, being inbred, or damage in transport), or they may not be released into the right locations at the right time. Such effects can be easily incorporated into our models, at least in a simple way: if released males have, say, one-tenth of the reproductive success of wild males, then the release rates calculated here need to be multiplied by 10. Incorporating low vigour due to laboratory adaptation may be somewhat more complex, as those traits would persist with the YLE for some generations. Most of our calculations have also assumed equal numbers released in each generation, but this may not be optimal. For example, it is possible that transportation costs are such that it will be better to release every second generation, or some other pattern. The fact that YLEs and their effects persist in the population better than other self-limiting constructs (figure 4) may allow greater operational flexibility. And as with many genetic strategies, impacts are determined by proportions, and, all else being equal, releasing into a small population will be more effective than releasing the same absolute number into a larger one. If the target population reproduces throughout the year, but shows seasonal cycles of abundance, then it may be most efficient to focus releases in the low season.

### (a) Molecular possibilities

It may be possible to develop synthetic constructs with properties much like those modelled here in many different ways. First, one might insert onto the Y chromosome an editor that knocks out a gene that is haplo-insufficient for viability or fertility and is X-linked or has female-limited effects. If the target gene is autosomal and affects both sexes, then a ‘rescue' copy of the gene might be inserted on the Y that is sufficiently diverged in sequence that it is not recognized by the editor and, if the editor is a nuclease, could not act as a template for homologous repair. Knock-outs can be produced by sequence-specific cleavage followed by end joining, in which case it may be useful to target sequences between direct repeats where micro-homology-mediated end joining would produce a frameshift mutation [33]. Conceivably one could insert a non-functional copy of the gene on the Y, to allow homologous repair, but it is not clear if this would be useful. Alternatively, instead of using a nuclease, one could use a base editor to, for example, change a C to a T and thereby introduce a premature stop codon [34,35]. In principle, YLEs could also be used to introduce dominant negative changes into a gene that is haplo-sufficient for survival or fertility. Again, the target gene would need to be X-linked or have female-limited effects, or there would need to be a rescue copy on the Y.

Second, the YLE might target the X chromosome in a number of other ways. It might cleave a sequence on the X, followed by homologous repair from the Y that inserts a female-specific dominant lethal gene. In some species inserting a copy of the male-determining gene may be sufficient [19,20]. This approach requires some homology between X and Y, which could pre-exist (e.g. in a pseudo-autosomal region) or could be engineered. Alternatively, the YLE might encode a protein that binds to the X and is carried with it into the zygote, where it causes death—a poison tag system that may, for example, use chromatin regulators [36,37].

Third, several possibilities arise if there can be paternal carry-over of the editing complex via the sperm into the zygote, as has been observed for the PpoI meganuclease in *Anopheles gambiae* mosquitoes [5,38]. For example, an editor might simply knock out a haplo-sufficient gene (rather than having to introduce a dominant negative edit) if, in addition to acting in the male germline, it also acts in the zygote to disrupt the maternally derived allele. Alternatively, paternal deposition could be used to target RNAs or proteins in the zygote. In many insects the sex determining pathway involves male- and female-specific alternative splicing of RNAs, and paternal deposition of a male-determining protein, or other molecule that can interact with this pathway, may be sufficient to disrupt female development while having little or no effect on males. More generally, a Y-linked spermatogenically expressed toxin and zygotically expressed antidote, analogous to those developed for synthetic underdominance and medea systems [39–41], could be used. A paternally active toxin–antidote system has been discovered in natural populations of the nematode *Caenorhabditis elegans* [42].

Our modelling also suggests that an autosomal construct showing dominant lethality in females and drive in males [18,21–24] can be as efficient as a YLE. Such an approach may be particularly appropriate in hermaphrodites that have no sex chromosomes. In the future it will be useful to compare these different molecular alternatives in terms of other criteria, including evolutionary stability in the face of mutations likely to arise after release [21,43].

Regardless of what precise mechanism is used, control sequences will need to be chosen to ensure the YLE is active in the male germline. If the edit has female-limited effects then some somatic expression may be acceptable, but if the target is on the X and there is no rescue copy on the Y then somatic expression may be harmful to the male and should be minimized. Similarly, expression may need to be as late as possible in the male germline if the target gene is expressed there. In some species the sex chromosomes are silenced around the time of meiosis, in which case expression may need to occur earlier (e.g. [44]) or the silencing circumvented. Rearing the organisms for release is likely to be easier if the YLE can be made repressible in the production facility [18]. It will also be important to consider the potential for resistance to evolve [33,45,46].

The effect of a YLE depends on its frequency in a population, which can be controlled in more ways than simply by adjusting the release rates. In some circumstances (e.g. early in a control programme) it might be desirable to purposefully introduce a fitness cost onto the modified Y (by adding harmful sequence or deleting non-essential sequences), which would both accelerate the rate at which the YLE disappeared from a population once releases stopped and further reduce the impact on non-target populations. In other circumstances it may be necessary to enhance the efficacy of control, which can be done by combining a YLE with an autosomal X-shredder. Both meganuclease- and CRISPR-based X-shredders that act by targeting the rDNA repeat have been developed in *An. gambiae* [5,6]. A conditional CRISPR-based X-shredder that only acts in the presence of the YLE could be designed by separating the Cas9 and guide RNA genes, inserting one on the Y and the other on an autosome. The efficiency of these ‘augmented YLEs' could be further enhanced if the X-shredder itself increased in the population by the homing reaction, and if it homed into a sequence found only in the target population, then it need not spread appreciably to other populations. The homing site could be a minority allele (less than 50% frequency) in the target population and still be useful. Alternatively, efficiency could also be increased by adding a third locus that increases the transmission of the X-shredder, as has been shown with the ‘daisy drives' of Noble *et al*. [47], or by placing the X-shredder in a pseudo-autosomal region, where linkage with the YLE could be tighter than if it is on an autosome, though some mechanism may then be needed to prevent an inversion creating a driving Y. Finally, while we have framed our discussion in terms of an X-shredder, what matters is having a locus that distorts the transmission of the sex chromosomes, regardless of the underlying mechanism. As we have noted, in species where siblings compete more than random, early female lethality may give a survival advantage to brothers, and an autosomal editor with this effect could be used instead of an X-shredder.

Recent advances in molecular biology and genome editing are opening up new possibilities for the safe and effective control of harmful species. The species for which genetic approaches are being considered vary widely, from disease-transmitting mosquitoes to invasive mammals, and it is unlikely that the same approach will be appropriate in all cases. YLEs offer a unique combination of features that may be useful in some applications, particularly if efficient control is needed in some locations and is to be avoided in others. Whether YLEs are useful in any particular application, and the design criteria needed for success, will require more tactical models tailored to the biology of the target species.

## Supplementary Material

Supplementary Tables 1 - 3

## Data accessibility

Computer code is available from Dryad Digital Repository https://doi.org/10.5061/dryad.474j412 [48].

## References

[1] Alphey L, Benedict M, Bellini R, Clark GG, Dame DA, Service MW, Dobson SL 2010 Sterile-insect methods for control of mosquito-borne diseases: an analysis. Vector-Borne Zoonotic Dis. 10, 295–311. (10.1089/vbz.2009.0014) 19725763 PMC2946175

[2] Alphey L 2014 Genetic control of mosquitoes. Ann. Rev. Entomol. 59, 205–224. (10.1146/annurev-ento-011613-162002) 24160434

[3] Burt A 2014 Heritable strategies for controlling insect vectors of disease. Phil. Trans. R. Soc. B 369, 20130432 (10.1098/rstb.2013.0432) 24821918 PMC4024225

[4] Godfray HCJ, North A, Burt A 2017 How driving endonuclease genes can be used to combat pests and disease vectors. BMC Biol. 15, 81 (10.1186/s12915-017-0420-4) 28893259 PMC5594614

[5] Galizi R, Doyle LA, Menichelli M, Bernardini F, Deredec A, Burt A, Stoddard BL, Windbichler N, Crisanti A 2014 A synthetic sex ratio distortion system for the control of the human malaria mosquito. Nat. Commun. 5, 3977 (10.1038/ncomms4977) 24915045 PMC4057611

[6] Galizi R et al. 2016 A CRISPR-Cas9 sex-ratio distortion system for genetic control. Sci. Rep. 6, 31139 (10.1038/srep31139) 27484623 PMC4971495

[7] Hammond A et al. 2016 A CRISPR-Cas9 gene drive system-targeting female reproduction in the malaria mosquito vector *Anopheles gambiae*. Nat. Biotechnol. 34, 78–83. (10.1038/nbt.3439) 26641531 PMC4913862

[8] Beaghton A, Beaghton PJ, Burt A 2016 Gene drive through a landscape: reaction-diffusion models of population suppression and elimination by a sex ratio distorter. Theor. Popul. Biol. 108, 51–69. (10.1016/j.tpb.2015.11.005) 26704073

[9] Eckhoff PA, Wenger EA, Godfray HCJ, Burt A 2017 Impact of mosquito gene drive on malaria elimination in a computational model with explicit spatial and temporal dynamics. Proc. Natl Acad. Sci. USA 114, E255–E264. (10.1073/pnas.1611064114) 28028208 PMC5240713

[10] Fu G et al. 2010 Female-specific flightless phenotype for mosquito control. Proc. Natl Acad. Sci. USA 107, 4550–4554. (10.1073/pnas.1000251107) 20176967 PMC2826341

[11] Dhole S, Vella MR, Lloyd AL, Gould F 2017 Invasion and migration of spatially self-limiting gene drives: a comparative analysis. *Biorxiv* (10.1111/eva.12583)

[12] Marshall JM, Hay BA 2012 Confinement of gene drive systems to local populations: a comparative analysis. J. Theor. Biol. 294, 153–171. (10.1016/j.jtbi.2011.10.032) 22094363 PMC3260013

[13] Deredec A, Godfray HCJ, Burt A 2011 Requirements for effective malaria control with homing endonuclease genes. Proc. Natl Acad. Sci. USA 108, E874–E880. (10.1073/pnas.1110717108) 21976487 PMC3203790

[14] Carvalho DO, McKemey AR, Garziera L, Lacroix R, Donnelly CA, Alphey L, Malavasi A, Capurro ML 2015 Suppression of a field population of *Aedes aegypti* in Brazil by sustained release of transgenic male mosquitoes. PLoS Negl. Trop. Dis. 9, e0003864 (10.1371/journal.pntd.0003864) 26135160 PMC4489809

[15] Mains JW, Brelsfoard CL, Rose RI, Dobson SL 2016 Female adult *Aedes albopictus* suppression by *Wolbachia*-infected male mosquitoes. Sci. Rep. 6, 33846 (10.1038/srep33846) 27659038 PMC5034338

[16] Zhang DJ, Lees RS, Xi ZY, Bourtzis K, Gilles JRL 2016 Combining the sterile insect technique with the incompatible insect technique: III-Robust mating competitiveness of irradiated triple *Wolbachia*-infected *Aedes albopictus* males under semi-field conditions. PLoS ONE 11, e0151864 (10.1371/journal.pone.0151864) 26990981 PMC4798476

[17] Schliekelman P, Gould F 2000 Pest control by the release of insects carrying a female-killing allele on multiple loci. J. Econ. Entomol. 93, 1566–1579. (10.1603/0022-0493-93.6.1566) 11142283

[18] Thomas DD, Donnelly CA, Wood RJ, Alphey LS 2000 Insect population control using a dominant, repressible, lethal genetic system. Science 287, 2474–2476. (10.1126/science.287.5462.2474) 10741964

[19] Criscione F, Qi YM, Tu ZJ 2016 GUY1 confers complete female lethality and is a strong candidate for a male-determining factor in *Anopheles stephensi*. Elife 5, e19281 (10.7554/eLife.19281) 27644420 PMC5061544

[20] Krzywinska E, Dennison NJ, Lycett GJ, Krzywinski J 2016 A maleness gene in the malaria mosquito *Anopheles gambiae*. Science 353, 67–69. (10.1126/science.aaf5605) 27365445

[21] Burt A 2003 Site-specific selfish genes as tools for the control and genetic engineering of natural populations. Proc. R. Soc. Lond. B 270, 921–928. (10.1098/rspb.2002.2319) PMC1691325 12803906

[22] KaramiNejadRanjbar M, Eckermann K, Ahmed HMM, Sánchez CHM, Dippel S, Marshall JM, Wimmer EA 2018 Consequences of resistance evolution in a Cas9-based sex-conversion suppression gene drive for insect pest management. Proc. Natl Acad. Sci. USA 115, 6189–6194. (10.1073/pnas.1713825115) 29844184 PMC6004448

[23] Adelman ZN, Tu ZJ 2016 Control of mosquito-borne infectious diseases: sex and gene drive. Trends Parasitol. 32, 219–229. (10.1016/j.pt.2015.12.003) 26897660 PMC4767671

[24] Piaggio AJ et al 2017 Is it time for synthetic biodiversity conservation? Trends Ecol. Evol. 32, 97–107. (10.1016/j.tree.2016.10.016) 27871673

[25] Schliekelman P, Ellner S, Gould F 2005 Pest control by genetic manipulation of sex ratio. J. Econ. Entomol. 98, 18–34. (10.1093/jee/98.1.18) 15765662

[26] Alphey N, Bonsall MB 2014 Interplay of population genetics and dynamics in the genetic control of mosquitoes. J. R. Soc. Interface 11, 20131071 (10.1098/rsif.2013.1071) 24522781 PMC3928937

[27] Phuc H et al. 2007 Late-acting dominant lethal genetic systems and mosquito control. BMC Biol. 5, 11 (10.1186/1741-7007-5-11) 17374148 PMC1865532

[28] Yakob L, Alphey L, Bonsall MB 2008 *Aedes aegypti* control: the concomitant role of competition, space and transgenic technologies. J. Appl. Ecol. 45, 1258–1265. (10.1111/j.1365-2664.2008.01498.x)

[29] Prout T 1978 Joint effects of release of sterile males and immigration of fertilized females on a density regulated population. Theor. Pop. Biol. 13, 40–71. (10.1016/0040-5809(78)90035-7) 644519

[30] Wolff JN, Gemmell NJ, Tompkins DM, Dowling DK 2017 Introduction of a male-harming mitochondrial haplotype via ‘Trojan Females’ achieves population suppression in fruit flies. Elife 6, e23551 (10.7554/eLife.23551) 28467301 PMC5441865

[31] Hurst GDD, Frost CL 2015 Reproductive parasitism: maternally inherited symbionts in a biparental world. Cold Spring Harbor Perspect. Biol. 7, a017699 (10.1101/cshperspect.a017699) PMC4448626 25934011

[32] Friberg U, Rice WR 2015 Sexually antagonistic zygotic drive: a new form of genetic conflict between the sex chromosomes. Cold Spring Harbor Perspect. Biol. 7, a017608 (10.1101/cshperspect.a017608) PMC4355276 25573714

[33] Hammond AM et al. 2017 The creation and selection of mutations resistant to a gene drive over multiple generations in the malaria mosquito. PLoS Genet. 13, e1007039 (10.1371/journal.pgen.1007039) 28976972 PMC5648257

[34] Billon P, Bryant EE, Joseph SA, Nambiar TS, Hayward SB, Rothstein R, Ciccia A 2017 CRISPR-mediated base editing enables efficient disruption of eukaryotic genes through induction of STOP codons. Mol. Cell 67, 1068–1079.e4. (10.1016/j.molcel.2017.08.008) 28890334 PMC5610906

[35] Komor AC, Zhao KT, Packer MS, Gaudelli NM, Waterbury AL, Koblan LW, Kim YB, Badran AH, Liu DR 2017 Improved base excision repair inhibition and bacteriophage Mu Gam protein yields C:G-to-T: a base editors with higher efficiency and product purity. Sci. Adv. 3, eaa04774 (10.1126/sciadv.aao4774) PMC5576876 28875174

[36] Keung AJ, Joung JK, Khalil AS, Collins JJ 2015 Chromatin regulation at the frontier of synthetic biology. Nat. Rev. Genet. 16, 159–171. (10.1038/nrg3900) 25668787 PMC4846386

[37] Park M, Keung AJ, Khalil AS 2016 The epigenome: the next substrate for engineering. Genome Biol. 17, 183 (10.1186/s13059-016-1046-5) 27582168 PMC5006378

[38] Windbichler N, Papathanos PA, Crisanti A 2008 Targeting the X chromosome during spermatogenesis induces Y chromosome transmission ratio distortion and early dominant embryo lethality in *Anopheles gambiae*. PLoS Genet. 4, 1 000 291–1 000 299. (10.1371/journal.pgen.1000291) PMC2585807 19057670

[39] Chen CH, Huang HX, Ward CM, Su JT, Schaeffer LV, Guo M, Hay BA 2007 A synthetic maternal-effect selfish genetic element drives population replacement in *Drosophila*. Science 316, 597–600. (10.1126/science.1138595) 17395794

[40] Akbari OS, Chen C-H, Marshall JM, Huang H, Antoshechkin I, Hay BA 2014 Novel synthetic medea selfish genetic elements drive population replacement in *Drosophila*; a theoretical exploration of medea-dependent population suppression. ACS Synth. Biol. 3, 915–928. (10.1021/sb300079h) 23654248 PMC3742681

[41] Akbari OS, Matzen KD, Marshall JM, Huang H, Ward CM, Hay BA 2013 A synthetic gene drive system for local, reversible modification and suppression of insect populations. Curr. Biol. 23, 671–677. (10.1016/j.cub.2013.02.059) 23541732 PMC8459379

[42] Seidel HS, Ailion M, Li JL, van Oudenaarden A, Rockman MV, Kruglyak L 2011 A novel sperm-delivered toxin causes late-stage embryo lethality and transmission ratio distortion in *C. elegans*. PLoS Biol. 9, e1001115 (10.1371/journal.pbio.1001115) 21814493 PMC3144186

[43] Beaghton A, Hammond A, Nolan T, Crisanti A, Godfray HCJ, Burt A 2017 Requirements for driving antipathogen effector genes into populations of disease vectors by homing. Genetics 205, 1587–1596. (10.1534/genetics.116.197632) 28159753 PMC5378115

[44] Bernardini F, Galizi R, Menichelli M, Papathanos PA, Dritsou V, Marois E, Crisanti A, Windbichler N 2014 Site-specific genetic engineering of the *Anopheles gambiae* Y chromosome. Proc. Natl Acad. Sci. USA 111, 7600–7605. (10.1073/pnas.1404996111) 24821795 PMC4040617

[45] Champer J, Reeves R, Oh SY, Liu C, Liu JX, Clark AG, Messer PW 2017 Novel CRISPR/Cas9 gene drive constructs reveal insights into mechanisms of resistance allele formation and drive efficiency in genetically diverse populations. PLoS Genet. 13, e1006796 (10.1371/journal.pgen.1006796) 28727785 PMC5518997

[46] Beaghton A, Beaghton PJ, Burt A 2017 Vector control with driving Y chromosomes: modelling the evolution of resistance. Malar. J. 16, 286 (10.1186/s12936-017-1932-7) 28705249 PMC5513332

[47] Noble C (2016).

[48] Burt A, Deredec A 2018 Data from: Self-limiting population genetic control with sex-linked genome editors. Dryad Digital Repository. (10.5061/dryad.474j412)

