# Supplementary material for: Self-limiting population genetic control with sex-linked genome editors
Source: Proc Biol Sci. 2018 Jul 25;285(1883):20180776. doi: 10.1098/rspb.2018.0776 (PMC6083257; doi:10.1098/rspb.2018.0776)
Supplement: Supplementary Tables 1 - 3 [file rspb20180776supp1.pdf]

Supplementary Information for:

Self-limiting population genetic control with sex-linked genome editors

Austin Burt [1]

Anne Deredec [2]

The Model

Genetics and fitness effects

The multiple diverse strategies are investigated with three genetic models. In the first, there are two Y chromosomes, wild-type and transgenic, denoted y and Y, and an autosomal locus with two alleles, wildtype and variant (mutant or transgenic, depending on the scenario), denoted a and A. There are thus 3 female genotypes (a/a, a/A, and A/A), producing 2 types of egg (a and A), and 6 male genotypes (ya/a, ya/A, yA/A, Ya/a, Ya/A, and YA/A), producing 4 types of sperm (ya, yA, Ya, YA). In a/A heterozygotes, the A allele is transmitted to a fraction *df* of eggs and *dm* of sperm (*df*=*dm*=0.5 for Mendelian transmission). In a/A males the Y chromosome is transmitted to a fraction *m1* of progeny, and in A/A males to a fraction *m2* of progeny (*m1*=*m2*=0.5 for Mendelian transmission). In males with a transgenic Y chromosome and at least one a allele at the autosomal locus, the a allele is converted to A with probability *u*; these conversions are assumed to occur in the germline of the male and have no effect on his survival or fertilization success. Table SI-1 shows the parameter settings for the various strategies, and Table SI-2 shows the proportions of gametes produced by the different genotypes.

In the second model, used for a YLE targeting an X-linked locus, the second locus is on the X chromosome instead of an autosome, with two alleles, wildtype and variant, denoted x and X. There are 3 female genotypes (x/x, x/X, and X/X), producing 2 types of egg (x and X), and 4 male genotypes (yx, yX, Yx and YX), producing 4 types of sperm (x, X, y and Y). Transmission is Mendelian except for the mutations of x to X in the presence of Y (Table SI-3). To incorporate an X-shredder, a third (autosomal) locus is added to the model, which determines the transmission rates of the sex chromosomes.

Population biology and selection

We model a population with discrete, non-overlapping generations. In the pre-intervention

wildtype population each generation begins with a certain number of hatchlings, denoted N_h. Non-selective density-dependent mortality occurs during the juvenile phase, such that the probability of surviving is equal to $\theta_J \alpha / (\alpha + N_h[t])$, where θ_J is the density-independent (or low density) probability a hatchling survives to become an adult; α is a constant determining the intensity of density-dependent mortality; and $N_h[t]$ is the number of hatchlings (male plus female) at time t. Individuals then become adults and mate randomly, with each wildtype female producing *f* fertilised eggs to start the next generation. Each juvenile is derived from an independent mating event, and males are assumed not to be limiting in the production of fertilized eggs. The intrinsic rate of increase of the population is

$$\mathrm{R}_m = f\theta_J/2$$

and the pre-intervention equilibrium population size is

$$\alpha \frac{(\mathrm{R}_m - 1)}{f} \qquad .$$

Selection on the autosomal or X-linked locus (i.e., differential survival or reproduction by genotype) can occur in one of three ways: (i) differential survival of hatchlings before the density-dependent mortality; (ii) differential survival of pre-adults after the density-dependent mortality; or (iii) differential fecundity of adult females and mating success of adult males. Whichever of these occurs, the fitness of wildtype genotypes (a/a or x/x) is standardised to 1; the fitness of heterozygotes (a/A or x/X) is $1 - h_f s_f$ for females and $1 - h_m s_m$ for males; and the fitness of homozygotes (A/A or X/X) or hemizygotes (X males) is $1 - s_f$ for females and $1 - s_m$ for males, where s_f and s_m are selection coefficients and h_f and h_m are dominance coefficients. In addition, males with a transgenic Y have fitness reduced by a factor $1 - s_Y$; for simplicity this selection assumed to occur at the same time and in the same manner as selection on the autosomal or X-linked locus. Key parameters differentiating the alternative genetic control strategies are shown in Table SI-1, idealized parameter values in Table SI-2, and fitnesses of the different genotypes shown in Tables SI-3 and SI-4.

Results: further details

Effect of halting releases

In the idealized case of a YLE with no effect on male fitness, halting releases stops the population decline, but it does not recover, and the population remains suppressed indefinitely (Main text, Fig. 4b). With a YLE-a the equilibrium abundance of females in our model is:

$$\frac{R_m(1-p)-1}{R_m-1} \qquad (1)$$

where p is the proportion of males with the YLE. Note that if $p > 1\text{-}1/R_m$ the population is eventually eliminated without further releases. Alternatively, if the YLE imposes a small fitness cost on males ($s_Y > 0$), then it will be slowly lost after releases are stopped, and the population slowly recover (Main text, Fig. 4c). In this case it can be shown that recurrent

release rates (η) above a threshold value

$$\eta > \mathrm{s}_Y \times \frac{\left(\sqrt{\mathrm{R}_m}-1\right)^2}{\mathrm{R}_m-1}$$

will lead to eventual elimination, whereas for release rates below this threshold the equilibrium frequency of a YLE-a is:

$$p = \frac{2\eta}{(\mathrm{s}_Y+\eta)+\sqrt{(\mathrm{s}_Y-\eta)^2-4\frac{\eta \mathrm{s}_Y}{\mathrm{R}_m-1}}} \quad ,$$

with the equilibrium abundance of females given by equation (1).

Table SI-1: Key parameters differentiating alternative genetic control strategies.

Strategy	Output from transgenic male x wildtype female crosses			
	Progeny sex ratio	Fitness of female progeny	Fitness of male progeny	Transgene transmission by male progeny
SIT / bi-RIDL	1:1	0	0	n/a
fs-RIDL	1:1	0	1	1:1
fs-RIDL-drive	1:1	0	1	All transgenic
X-shredder	All male	1	1	1:1
YLE	1:1	0	1	1:1 (male limited)

Table SI-2. Idealised parameter settings for alternative genetic control strategies.

Strategy	Transmission rates				Mutation rate (u)	Fitness parameters					Genotype released
	d_m	d_f	m_1	m_2		h_f	s_f	h_m	s_m	s_Y	
bi-RIDL	1/2	1/2	1/2	1/2	0	1	1	1	1	-	yA/A
fs-RIDL	1/2	1/2	1/2	1/2	0	1	1	0	0	-	yA/A
fs-RIDL-drive	1	1	1/2	1/2	0	1	1	0	0	-	ya/A
X-shredder	1/2	1/2	1	1	0	0	0	0	0	-	yA/A
Y-linked editor	1/2	1/2	1/2	1/2	1	1	1	0	0	0	Ya/a

d_m and d_f are transmission rates of the A allele in a/A heterozygous males and females, respectively. m_1 and m_2 are transmission rates of the Y chromosome (whether transgenic or not) in a/A and A/A males, respectively. u is the rate at which a alleles mutate to A in transgenic Y males. s_f and s_m are selection coefficients against homozygous A/A females and males, h_f and h_m are the corresponding dominance coefficients, and s_Y is the selection coefficient against the Y-linked editor gene.

Table SI-3. The fitness of each genotype and the proportion of each gamete produced by them for the model of polymorphic Y chromosome and autosome

Diploid genotype	Fitness	Gametes produced					
		xa	xA	ya	yA	Ya	YA
xaa	1	1	0	0	0	0	0
xaA	$1-h_f s_f$	$1-d_f$	d_f	0	0	0	0
xAA	$1-s_f$	0	1	0	0	0	0
yaa	1	1/2	0	1/2	0	0	0
yaA	$1-h_m s_m$	$(1-d_m)(1-m_1)$	$d_m(1-m_1)$	$(1-d_m) m_1$	$d_m m_1$	0	0
yAA	$1-s_m$	0	$1-m_2$	0	m_2	0	0
Yaa	$1-s_Y$	$(1-u)/2$	$u/2$	0	0	$(1-u)/2$	$u/2$
YaA	$(1-s_Y)(1-h_m s_m)$	$(1-u)/4$	$(1+u)/4$	0	0	$(1-u)/4$	$(1+u)/4$
YAA	$(1-s_Y)(1-s_m)$	0	1/2	0	0	0	1/2

Table SI-4. The fitness of each genotype and the proportion of each gamete produced by them for the model of polymorphic Y and X chromosomes

Diploid genotype	Fitness	Gametes produced			
		x	X	y	Y
xx	1	1	0	0	0
xX	$1-h_f s_f$	1/2	1/2	0	0
XX	$1-s_f$	0	1	0	0
yx	1	1/2	0	1/2	0
yX	$1-s_m$	0	1/2	1/2	0
Yx	$1-s_Y$	$(1-u)/2$	$u/2$	0	1/2
YX	$(1-s_Y)(1-s_m)$	0	1/2	0	1/2

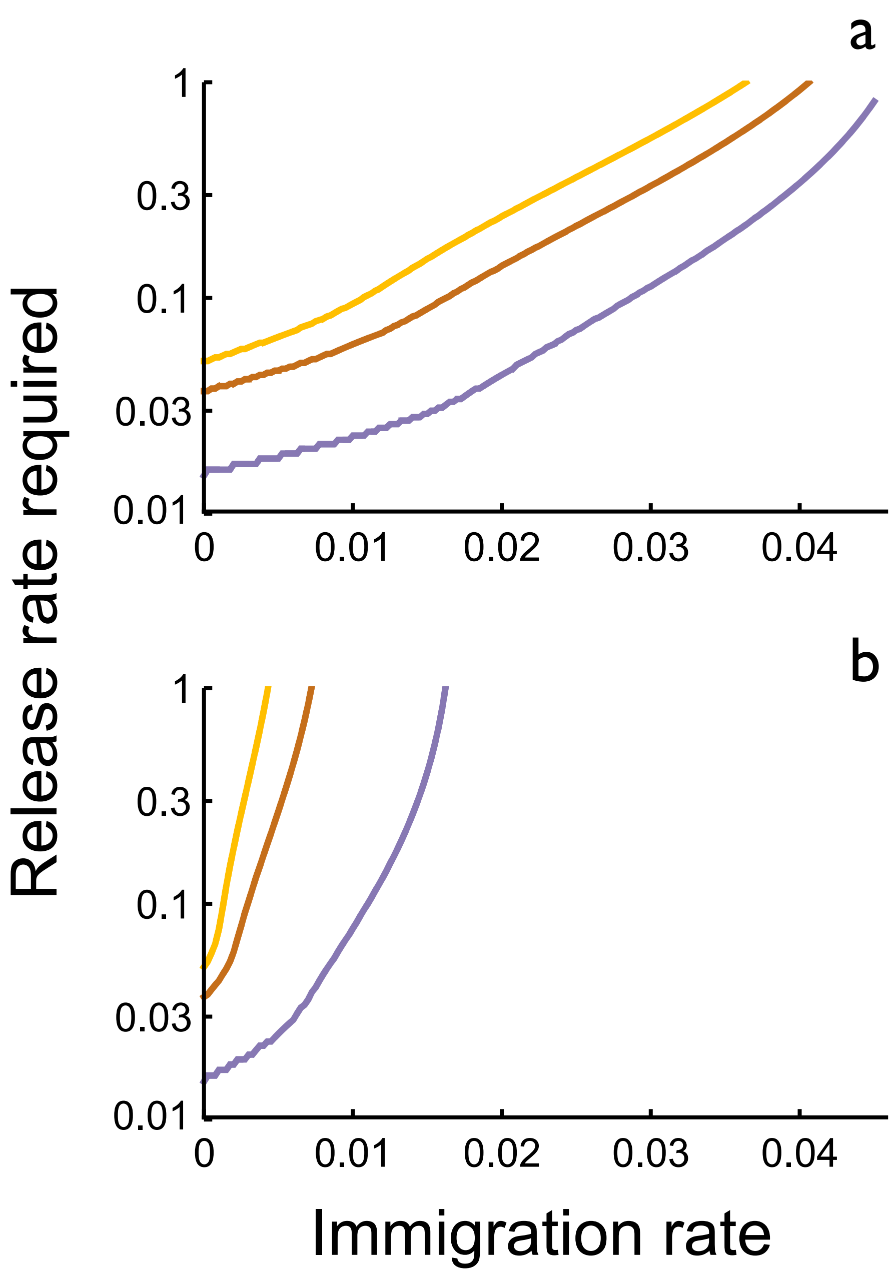

Fig. SI-1. Release rates required to suppress the number of females in a target population to 5% of its initial value in 36 generations with a YLE-a construct as a function of the immigration rate, for Rm = 2, 6, and 12 (purple, brown and yellow, respectively). Immigrants are adult males and unmated females (a) or mated females (b). Results are the same whether target locus is autosomal or X-linked. We assume here that movement is unidirectional, from non-target to target populations, so all immigrants are wildtype (also equivalent to assuming the non-target population is very large). As expected, higher rates of immigration mean higher release rates of transgenics are needed to achieve control, and if the number of immigrants each generation is equal to a proportion g of the initial population size, then no matter how many males of whatever genotype are released, it will not be possible to reduce the population below g (unless immigration rates or the source population are also controlled). Immigration of mated females presents more of a problem than immigration of unmated females.

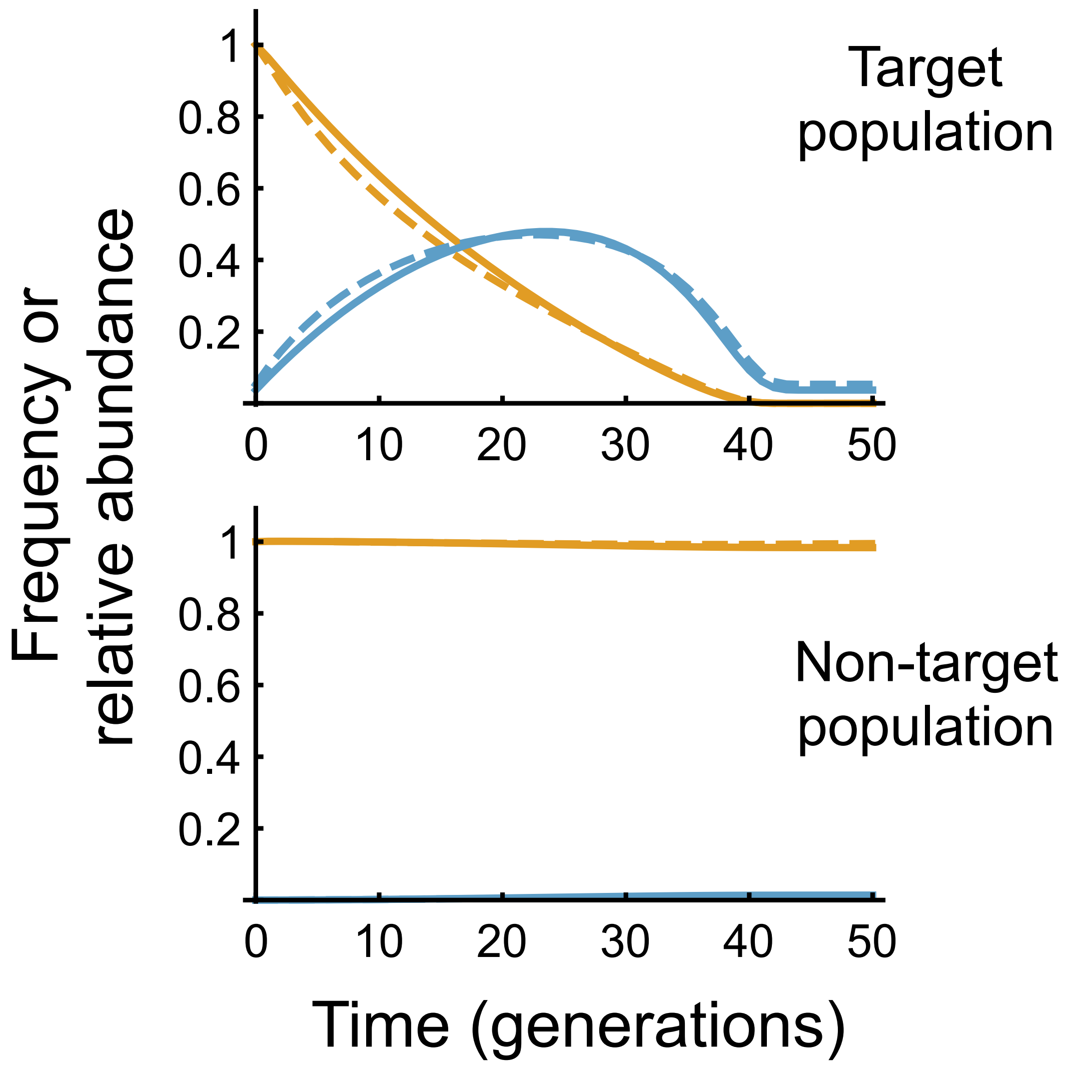

Fig. SI-2. Frequency of YLE males (blue) and relative numbers of females (orange) in target and non-target populations over time. Solid lines show no cost of the YLE (sY=0) with a release rate (that continues for the full duration of the simulation) of 3.7%, and dashed lines show sY=0.05 with release rate of 5.3% — in both cases the release rates are sufficient to give 95% suppression in 36 generations. In every generation a pre-reproductive (and pre-mating) adult has a probability of 0.1% of moving from the target to non-target populations, regardless of gender or genotype. The two populations are assumed to be the same size and, for simplicity, migration in the opposite direction is ignored.
